# The migration-related language barrier and professional interpreter use in primary health care in Switzerland

**DOI:** 10.1186/s12913-019-4164-4

**Published:** 2019-06-27

**Authors:** Fabienne N. Jaeger, Nicole Pellaud, Bénédicte Laville, Pierre Klauser

**Affiliations:** 1 Kollegium für Hausarztmedizin, Bern, Switzerland; 2 Swiss Tropical and Public Health Institute, University of Basel, Basel, Switzerland; 3 Swiss Society of Paediatrics, Fribourg, Switzerland; 4 Kollegium für Hausarztmedizin (KHM), Rue de l’Hôpital 15, CH-1701 Fribourg, Switzerland; 5 Swiss Tropical and Public Health Institute, Socinstrasse 57, CH-4002 Basel, Switzerland

**Keywords:** Language barrier, Interpreter, Primary care, Paediatric, Family doctor, Migrant, General practitioner

## Abstract

**Background:**

With increased international migration, language barriers are likely becoming more relevant in primary care. The aim of this study was to investigate the language barrier in paediatric and adult primary care, present its consequences, reveal how it is overcome, as well as highlight the use of and potential unmet needs for professional interpreters, using Switzerland as a case study.

**Methods:**

Primary healthcare providers were invited nation-wide to participate in an online questionnaire on language barriers faced and interpreter use.

**Results:**

More than 90% of the 599 participants in this nation-wide cross-sectional study face relevant language barriers at least once a year, 30.0% even once a week. Using family members and friends for translations is reported as the most frequent resort for overcoming the language barrier (60.1% report it for more than 50% of encounters), followed by “using gestures” (32.0%) or just accepting the insufficient communication (22.9%). Minors interpret frequently (frequent use: 23.3%). Two thirds of physicians facing language barriers never have access to a professional interpreter, the majority (87.8%) though would appreciate their presence and approximately one quarter of these even see a cost-saving potential. Multiple consequences affecting quality of care in the absence of professional interpreters are identified.

**Conclusion:**

Language barriers are relevant in primary care. Improved access to professional interpreters is warranted.

## Background

With increased migration, healthcare providers in host countries may need to care more frequently for patients with whom they do not share a common language [1]. In 2017, continental Europe hosted 2.6 million refugees and nearly 1 million asylum-seekers [2]. Non-forced international migration further increases the variety of languages. For example, Eurostats estimates 16.9 million European Union (EU) citizens live in another EU country, and the number of non-EU citizens in the EU at 21.6 million, with the number of foreign permanent residents varying greatly from one country to the other [3]. In Switzerland, a country situated in the heart of Europe, which we shall use as a case study, the variety of languages and cultures is further accentuated by foreign permanent residents (24.9%) [4], of which the majority (68%) originate from the EU-28/EFTA countries [5], and migrants in the asylum process. Most asylum seekers originate from Eritrea, Afghanistan, and Syria, followed by Somalia, Sri Lanka, Iraq, Nigeria and the Gambia [5]. While people originating from neighbouring countries and qualified foreign employees (“expats”) are normally able to express themselves in a national language or English, other migrants may, especially upon arrival, face language barriers, including when seeking care.

In a study investigating challenges to family doctors providing care to international migrants in central Switzerland, the language barrier was considered the second most challenging part after the patients’ psycho-social problems [6]. Communication is central to patient-doctor encounters: doctors need to be able to take an adequate history in order to guide diagnoses; explanations of treatments, preventive aspects and further care need to be understood by patients and care-givers. The more delicate, complex and emotional the topic, the higher a language proficiency is required for adequate care [7].

The international literature indicates that the presence of professional interpreters can improve quality of care [8]: Reductions of unnecessary and potentially harmful exams, treatments and hospitalisations [9], increased adherence and use of preventive measures [8, 10], reduced durations of hospitalisations and needs for re-hospitalisations [11], and fewer adverse events [8] are advantages of professional interpreter interventions. Furthermore, they increase patients’ satisfaction with the encounter [12, 13] and may help clarify cultural aspects [14]. With adequate communication being the basis of quality medical care, the use of professional interpreters is generally recommended for bridging a language gap in medical encounters [8].

Still, in reality, the staff of facilities and patients friends’ and family members, sometimes even minors, interpret, thus rend a spoken message from one language to the other [15–18], this despite poorer quality of interpreting and potentially negative consequences [8]. Health facilities may benefit from bilingual staff able to consult directly [19]. If perfectly bilingual, such medical encounters yield better patient recall and allow patients to ask more questions [20]. Some hospitals can rely on a pool of multilingual staff to step in for short interpretations, may provide lists of multi-lingual staff members and provide training for them [21]. Such ad hoc interpretations by bilingual staff appear attractive in hospitals when they are easily available and sometimes may even appear of sufficient quality in the eyes of physicians [22, 23]. Still, if such bilingual staff is not trained in interpreting, problems regarding quality of interpreting and confidentiality, as well as role conflicts may arise, and the staff may be missing for other tasks [24]. The better the interpreter training, the fewer translation errors with potentially negative consequences are registered [25, 26]. In decentralised primary care settings with small providers, the staff pool to rely on for interpreting is more limited.

Non-hospital based primary healthcare in Switzerland, for example, is mainly provided on a private bases by family doctors, also called general practitioners, who predominantly see adults but sometimes also children and adolescents, and paediatricians in small private practices. Both undergo nationwide accredited specialist training before being allowed to operate on their own. Hospitals provide emergency services and hospital-based care, but are also available for ambulatory surgeries and specialist consultancies. Health insurance is mandatory and also covers asylum-seekers, thus migrants who have applied for asylum and are awaiting a decision, and refugees, thus migrants who applied for asylum and are, at least for the moment, allowed to stay, but it does not pay for interpreting services. 18 regional centers covering the entire country coordinate requests for professional interpreter services on site, the national telephone interpreter service provides interpreter services in more than 50 languages every day twenty-four hours a day at a cost [27]. Professional interpreters are usually accredited and though sometimes subsidized most often come at a cost to the medical institution requesting their service [27]. Public hospitals often receive funds or budget ahead to pay for expenses related to interpreter use [21, 28]. Still, language barriers have been identified as problematic to patient care in the hospital-based setting in Switzerland [29, 30].

Swiss private practices cannot usually rely on such funds and their staff pool of which to recruit ad hoc interpretations from is very small. Despite likely language barriers, they provide essential care. Family doctors and primary care paediatricians are the first-line curative and preventive health care providers, thus playing an essential central role in the health care system, reason why we focus on them. While research nationally [22, 23] and internationally [11, 17] focus mainly on the hospital setting, relevant data to inform policies on interpreter services in primary health care are lacking in Europe and in Switzerland particularly. The aim of this study was therefore to investigate i) the extent of the language barrier in Swiss non-hospital-based adult and paediatric primary care practices, ii) how it is overcome, ii) the use of professional interpreters and iii) a potential gap in access and use of interpreter services and iv) consequences thereof.

## Methods

We sent an online questionnaire to as many primary care physicians as possible in all of Switzerland. The questionnaire was developed in German based on a review of the literature, personal experience by the authors and enquiries to fellow primary care physicians. It was professionally translated to French and Italian and programmed using an online survey tool. It was piloted on a small number of primary care practitioners. It contained a maximum of 34 questions (including free text specifications) presented when relevant according to previous answers and took between 5 and 15 min to complete depending on answers given. Main areas covered where frequency of consultations with language barriers and how these are addressed, frequency of interpreter use, unmet interpreter needs and encountered consequences. The presented data are part of a larger study. The questionnaire can be accessed in the appendix.

We aimed at reaching the best possible national coverage in order to provide answers representative for Switzerland regarding language barrier and interpreter use in the non-hospital based general adult and paediatric primary care setting, thus catching the perspective of family doctors (FD) and primary care paediatricians (PCP). Physicians working in both settings – hospital and non-hospital primary care - were asked to only consider their non-hospital activities for the purpose of this questionnaire. The Swiss Society of Paediatricians (SSP) has 1020 members registered as working in paediatric primary ambulatory care; the association of Swiss family doctors and primary care paediatricians for political issues, Haus- und Kinderärzte Schweiz (MFE), has 4358 family doctors and 500 primary care paediatricians in its registry. Both societies operate nationwide with members in all regions. SSP and MFE each sent off two email invitations with an interval of two weeks in February and March 2017 to all their members. To avoid asking paediatricians who are members in both associations twice, known double members (*N* = 373) were first addressed by MFE with the reminder email being sent by SSP. Participation in the questionnaire was entirely voluntary and anonymous. Data were analysed descriptively and differences examined using Chi-square test in Stata IC 14. The study was not designed to demonstrate differences between paediatricians and family doctors, but whenever there was a significant difference results were presented separately for both groups.

The Swiss national ethics board confirmed that no specific ethic clearance was required.

The study was funded by the Kollegium für Hausarztmedizin (KHM, college of primary care), a foundation focusing on improving quality, prevention, research and education in Swiss primary care.

## Results

A total of 628 physicians participated in the questionnaire of which 29 had to be excluded as not working in non-hospital based primary care leaving 599 respondents. Thus the response rate was 11.6% with a higher response rate by PCP (25.2%) than FD (8.1%).351 (58.6%) respondents worked as FD and 247 as PCP. A participant working in non-hospital primary care who had failed to state if as family doctor or paediatrician was included in the overall analysis.

Good coverage of Switzerland was obtained with primary care physicians of all but one small region (half canton: 16000 inhabitants), participating. 71.3% replied in German, 25.9% in French and close to 3% in Italian, which indicates that Italian speakers may have been underrepresented, though some may have filled in a non-Italian version. One person indicated usually using Rhaeto-romanic, the fourth national language, which is only spoken by 0.5% of the population [31]. 45.2% of physicians work in a city, 29.6% in urban outskirts and 25.2% in the countryside. Men and woman participated equally (262 men, 50.1%).

### The language barrier

The vast majority of physicians (90.8%) report facing consultations with language barriers defined as the impossibility of a qualitative adequate direct communication due to language differences with the patient or – in the paediatric setting – the caregiver. Language barriers concern paediatricians more frequently than FD: PCP were four times less likely to never face such language barriers (3.6%) than FD. They were also more likely to face them at least 1x/week (36.8% vs. 26.8%) (See Table 1). There was no difference observed between rural or city practices (*p* = 0.32).

**Table 1:** Incidence of language barrier, interpreter use, unmet interpreter need and perceived costs saving potential

Frequency consultations with language barriers (hindering direct quality communication)									
*p* < 0.001 (FD vs. PCP)	N total	< 1x/ year		≥ 1x/year (<1x/month)		≥ 1x/month (<1x/week)		≥ 1x/week	
Total	599	55	9.2%	153	25.5%	206	34.3%	185	30.9%
FD	351	46	13.1%	87	24.8%	124	35.3%	94	26.8%
PCP	247	9	3.6%	66	26.7%	81	39.5%	91	36.8%
Frequency interpreter interventions^a^									
*p* = 0.003 (FD vs. PCP)	N total	< 1x/ year		≥ 1x/year (<1x/month)		≥ 1x/month (<1x/week)		≥ 1x/week	
Total	506	338	66.8%	120	23.7%	41	8.1%	7	1.4%
FD	286	210	73.4%	56	19.6%	17	5.9%	3	1.1%
PCP	219	127	58.9%	64	29.2%	24	11.0%	2	1.8%
Frequency interpreter desired but currently not present^a^									
*p* = 0.06 (FD vs. PCP)	N total	< 1x/ year		≥ 1x/year (<1x/month)		≥ 1x/month (<1x/week)		≥ 1x/week	
Total	501	61	12.2%	177	35.3%	196	39.1%	67	13.4%
FD	285	42	14.7%	105	36.8%	107	37.5%	31	10.9%
PCP	215	19	8.8%	72	33.5%	88	40.9%	36	16.7%
Frequency cost saving potential through additional interpreter use^a^									
*p* = 0.66 (FD vs. PCP)	N total	< 1x/ year		≥ 1x/year (<1x/month)		≥ 1x/month (<1x/week)		≥ 1x/week	
Total	423	138	32.6%	176	41.6%	92	21.8%	17	4.0%
FD	234	73	31.2%	104	44.4%	48	20.5%	9	3.9%
PCP	188	65	34.6%	72	38.3%	43	22.9%	8	4.3%

^a^ only concerns respondents facing language barriers

**Fig. 1:**
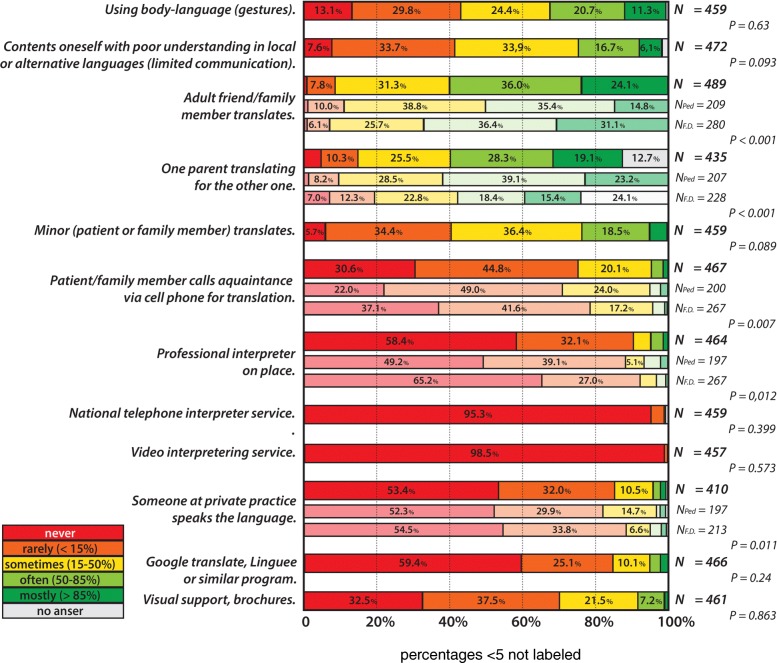
Adressing the language barrier: frequency of use

Only participants facing consultations with language barriers were presented with follow-up questions regarding coping with the language barrier, interpreter use and needs. Different strategies exist to address such language barriers. Participants were asked to estimate how often, in case of a real language barrier, they used which strategy to still try to communicate. Figure 1 shows the distribution of different frequencies of strategies used as reported by respondents.

As show in Fig. 1, the strategy participants use most often is interpreting by adult and family friends, but the use of gestures and body language, interpretations by minors or simply contenting themselves with the limited communication are also common. Almost all respondents use minors as interpreters at least some of the time. Regular use of brochures and visual aids to help bridge the language barrier is quite rare (8.2%).

Comparing between FD and PCP, the adult family members are more often the solution for FD (*p* = 0.001) whereas one parent translating for the other is more common with paediatricians (*p* < 0.001): 62.3% of PCP even report this for the majority of their consultations with a language barrier. Minors interpreting did not reach significant difference levels between the provider groups (*p* = 0.08). Paediatricians use professional interpreters more frequently (*p* = 0.012). More than 2/3 of FD and nearly half of PCP never use professional interpreters present in person, and if they are used, it tends to be rarely the case (for details see Fig. 1).

The national telephone interpreter service is very rarely used: 3.6% of respondents report using it in less than 15% of encounters with a substantial language barrier and only 3 PCP reporting more frequent use (3/464 respondents). Video interpreter service use was only confirmed by 6 out of 457 participants answering this question.

Online tools, patients calling acquaintances for interpretation via the telephone and members of the private practice helping out with interpretations also help bridge the language gap, but less frequently. 6 respondents added a comment that they rely on their multiple language skills (5–6 languages) to overcome the language barrier.

### Professional interpreter use

Professional interpreter use was further investigated, as it is considered providing the best quality of translations. Professional interpreter use was more frequently reported for paediatric consultations: Only 26.6% of FD vs. 41.1% of paediatricians report the intervention of a professional interpreter at least once a year over the past year. Paediatricians also reported at least monthly interventions nearly twice as often (12.8% vs. 7%) as FD.

When asked to report the frequency of professional interpreter interventions per year, 167 respondents who face language barriers reported an average of 16.8 interventions with a median of 5 interventions – this skewed distribution was due to a few participants who report more frequent interventions (p95 = 48), of which 6 reported more than 100 interpreter interventions a year. A participant explained this due to his activities as doctor responsible for asylum seekers assigned to him by the local authorities.

Professional interpreter interventions can either be initiated by the healthcare provider or a third party, such as asylum authorities or social services. 169 out of 492 participants (34.4%), confirm having benefited from professional interpreter services organised by a third party, this being more frequently the case for PCP (40.6%; 86/212) than FD (29.8%; 83/279, *p* = 0.04). Participants estimated that 57% (SD 41.07) of occurring professional interpreter interventions are initiated by a third party, 35.3% stating that all are externally organised.

Professional interpreter interventions initiated by a third party usually concern asylum seekers and refugees (100% of FD and 92.7% of PCP report these groups being among the beneficiaries) with the main share being asylum seekers. It is therefore not surprising that interventions are most frequently organised by asylum centres (PCP confirm having encountered interventions organised by them in 44.4%, FD in 35.4%), authorities (PCP 36.7%, FD 35.4%), and non-governmental organization NGOs (PCP8.9%, FD 21.5%) – this depending on who is responsible for the asylum seekers and refugees in an administrative region. On rare occasions, international companies and embassies also organise interpreters. 7.3% of PCP confirm this for companies, 6.1% for embassies, figures being slightly lower for FD (5.6 and 2.8%). Patients organising professional interpreter interventions themselves is rare.

44% of participants caring for language incongruent patients (224/498) have already organised professional interpreters themselves. This was significantly more frequently stated by PCP (54.7%, 117/213; *p* < 0.001) than FD (37.8%; 107/283). Adjusted for the overall study participants this corresponds to 47.4% of (117, 247) of PCP and 30.5% (107/315) of FD. Among them 79.0% state only having organised professional interpreters translating in person at their private practice, 4.9% only using telephone based interpreting services, and 16.1% both with the distribution being similar for PCP and FD.

### Existing gap in professional interpreter use

The majority of physicians caring for patients with a language barrier confirm an unmet need for interpreter services in their practice (87.8%; PCP: 91.2%, FD: 85.6%). Extrapolating these answers to all participants, thus even those not usually confronted with language barriers during their work, the perceived gap still remains high: 73.5% (440/599) claim facing unmet needs for professional interpreters at least once a year. This is especially true for PCP (79.5%) and to a lesser degree for FD (69.2%). Roughly 1/3 of all participants even confirm such a need 1-3x/month, 11% at least 1x/week, thus revealing that half of the participants would appreciate (additional) interpreter services at least once a month (see Table 1 for details). Among those facing consultations with language barriers, the median estimated amount of additional interpreter interventions desired was estimated at 12 per year with a mean of 15, this again due to a skewed distribution (p 75 = 36/year, p 95 = 120/year).

The overall estimated need, thus including currently existing and desired professional interventions lays at a mean of 35.7 professional interpreter interventions a year (median 15) among those facing language barriers, whereas the median of consultations with a relevant language barrier hindering direct communication was estimated at 24 encounters per year (very skewed distribution, mean 58.6/year for only concerned respondents, mean 46.1/year for overall participants). 48 interventions per year would cover the needs of approximately 75% of these providers.

The main gap in professional interpreter interventions concerned consultations of asylum seekers and, to a lesser degree, refugees (together 381/465, 81.9%, FD: 77.4%, PCP: 87.7%; *p* = 0.04). Non-asylum new arrivals were also considered in 38.3% (FD 33.7%, PCP: 44.3%; *p* = 0.04), and first generation migrants living in the country for a long time but not being language proficient enough for the complexity of medical encounters in 33.7% (FD: 37.2%; PCP 29.6%, *p* = 0.08) as groups that would need professional interpreter support.

### Perceived consequences of insufficient interpreter use

While professional interpreter interventions do come at a cost, they may also have a cost saving potential. Two thirds believe saving heath care costs through the use of professional interpreters would have been possible at least once a year (67.3%, 285/423), one forth even once a month (see Table 1).

**Fig. 2:**
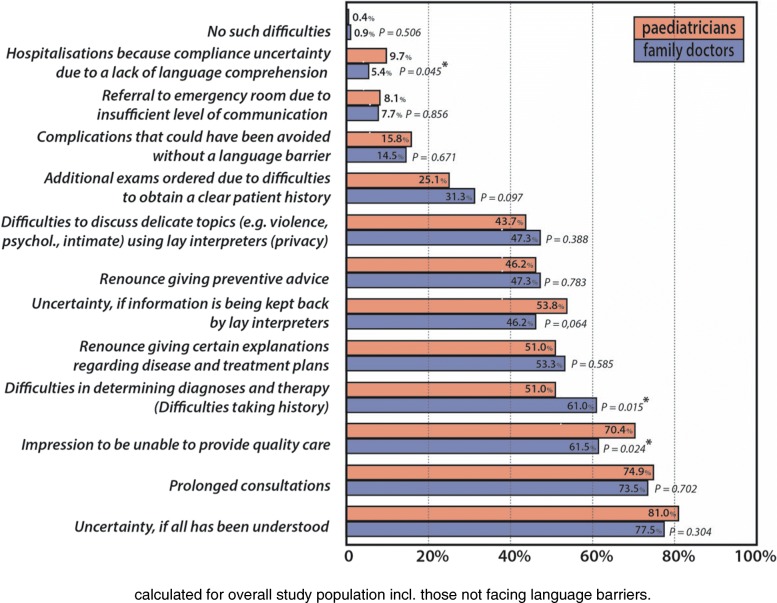
Difficulties due to language barriers encountered during the last year

Insufficient use of professional interpreters can lead to various situations with potentially negative consequences on quality of care (see Fig. 2, values given for overall participants). Participants were therefore presented with a list of situations potentially arising as a consequence of unaddressed language barriers and asked to indicate all those they had encountered at least once over the past year that could have been mitigated if a professional interpreter had been present. Of the 504 respondents answering these questions on potential consequences 4/5 of PCP and ¾ of FD felt they had not been able to provide appropriate care for patient and family due to the language barrier (total 77.6%, FD: 75.5%, PCP: 80.2%, *p* = 0.215; overall participants: 65.3%,). Nearly 2/3 of respondents (62.3%) reported difficulties determining the right diagnoses due to difficulties in obtaining a full patient history, with FD more often concerned hereof (FD: 74.8%, PCP: 58.1% of respondents, *p* < 0.001). It is therefore not surprising that they tend to confirm having ordered additional exams more often (FD: 38.5 vs. PCP: 28.6% of respondents, *p* = 0.021) due to an insufficient patient history. Extrapolated to all questionnaire participants, this would represent 28.9% of physicians having ordered extra exams at least once a year due to the language barrier.

Renouncing giving preventative advice was stated in 55.8% (overall participants: 46.9%), renouncing giving information on the disease, therapy and care plans in 62.3% (overall participants: 52.4%). Adverse events that could have been avoided through interpreter use were reported by nearly 1/5 (17.9%; overall participants: 15.0%). 11% of PCP and 6.6% of responding FD (*p* = 0.065) have already hospitalised patients (overall participants 7.2%; PCP: 9.7%, FD: 5.4%, *p* = 0.045) because of unsure compliance due to the language barrier. 9.3% of respondents have already sent patients to the emergency room as they could not communicate sufficiently well (overall participants: 7.8%). Increased duration of consultations was reported by 90% of respondents. Less than 1% of the 504 participants who replied to these questions never have encountered any such problems. Asked about intercultural challenges and problems, only 19.9% of PCP and 27.1% of FD reported never experiencing them.

## Discussion

This paper for the first time demonstrates the important extent of the language barrier encountered in Swiss non-hospital primary care. Asylum-seekers and refugees are most affected, but important language barriers also exist with other migrants. Adult lay interpreters are the most frequently used strategy to communicate in case of a language barrier. In their absence physicians mostly have to make do with body language and insufficient comprehension. Minors are also asked to interpret. Professional interpreters are occasionally used - especially in the asylum sector and more frequently by paediatricians than family doctors - but by far not according to identified needs. Even though physicians rarely use professional interpreters, they claim that their presence would help. They also affirm negative consequences due to the lack of professional interpreters that may impact quality of care. A substantial number of respondents even see a healthcare cost saving potential at least occasionally through the use of professional interpreters. The majority of respondents have also faced intercultural difficulties, some of which might be overcome with the help of professional interpreters.

Our data demonstrate a high reliance on patient family and friends for overcoming the language barrier in non-hospital primary health care. The use of friends and family to interpret is cost-free and often readily available [18]. Family and friends may sometimes be a source of support [32] and able to add valuable information [18]. However, the quality of their interpreting is usually inferior to that of professional interpreters because emotional cues tend to get interpreted to a lesser degree [33] and more translational errors with potentially harmful consequences and omissions occur [8, 25]. Loyalty conflicts and socio-cultural taboos (not able to state a severe diagnoses) may conflict with proper interpretation [34]. Furthermore, interpretations may be a source of embarrassment, e.g., for children who have to interpret discussions of intimate matters [34]. Interpreting can be emotionally hard on professional interpreters [35], but may be even harder on friends and family members, especially minors, depending on the issue at stake. The fact that minors also interpret frequently is therefore worrisome.

### Comparing with other settings

Comparing our findings with those from other settings, similar strategies used to overcome the language barrier as well as a generally limited interpreter use are noted. In a Swiss hospital based study [22] in a region with high numbers of foreigners, 71% of medical staff state having cared for patients nonproficient in the local language over the last 6 months and 51% of them confirmed having used a professional interpreter at least once. Great inter-department variety between medical disciplines was noted, despite the hospital covering interpreter costs. For comparison: between 62.1% (FD) to 76.3% (PCP) of respondents reported at least one consultation with a language barrier a month in our study and only 5,7 to 10.5% had used an interpreter once a month, but 39.3 to 50.2% would have liked the presence of an interpreter over the same time frame. In the investigated hospital more than 80% of healthcare providers stated having communicated directly with patients in another language than the local language, such as English, over the past 6 months, which is not surprising considering the multiple international organisations and companies in its catchment area [22].

While most international studies on interpreter focus on hospitals [17, 22, 23, 36], two studies from New Zealand [37] and one in Australia [38] also examined interpreter use in the primary healthcare setting. The Australian national study sampled general practitioners nationwide: 16.2% of consultations regarded patients speaking another language then the local language at home, a figure likely to be higher in Switzerland, 5% consultations involved speaking a foreign language, most of which were conducted by multilingual general practitioners and only a subgroup involved family members interpreting. Despite free-of-charge interpreter services available in Australia, interpreters were rarely used (1%), but physicians did see that their use would have improved 27.8% of such consultations [38]. Similar numbers of interpreter use were found in the analysis of general practice data of a region in New Zealand [37].

In a larger primary care facility caring for a high number of refugees (25%) and even having an in-house interpreter available, family members still interpreted in 49% of consultations, and their interpretation was judged to be good by staff, especially in case of on-the-day presentations [18]. Best ratings though were – also because providing a continuum allowing for the building of trust - received by the in-house interpreter available for 28% of consultations – a luxury non-existent in Switzerland where private practices are usually very small and patient groups too diverse to allow for interpreters for every language. In addition, phone interpreters were used in 21%. In this rather ideal setting, patient choice, borderline language proficiency, an underestimation of the language barrier, time constraints and oversight were stated as reasons for not using professional interpreters when their use would have been actually judged indicated. [18], Access to interpreters is less straightforward for most non-hospital primary care physicians in Switzerland: professional interpreters are accredited but nationwide funding of their services is lacking.

### Strengths and limitations

Participation may have been influenced by the perceived need for interpreters. Furthermore, participation rates (FD: 8.1%; PCP: 25.2%) were not very high, but good for a physician survey. Still, total numbers are sufficient to be representative with good power: an event with a probability of 50% could be determined with a 95% confidence interval and a margin of error of only app. 5.43% for the paediatricians being SSP members and 5.1% for the family doctors being member of MFE. Lower reply rates in FD may be explained through the link to the questionnaire having been sent via an online newsletter, while for SSP members this was in a separate email focusing on the questionnaire. More attractive placing of the link in the MFE-newsletter increased FD reply rates drastically. A shortfall in Italian speaking replies may be due to the MFE-newsletter being sent out in French and German, with the Italian minority choosing the language of their preference.

The study may be considered representative for members of SSP and MFE and for the country as both operate nationwide. It contributes to a better understanding of the extent of the language barrier, the ways primary care physicians cope with it and the existing gap in interpreter use.

The fact that paediatricians, who also participated more widely in the study, show higher rates of consultations with language barriers may be linked to the fact that elderly people migrate less frequently than younger ones. Already at the beginning of their stay in the new country, at a time when language proficiency has not been acquired yet, younger migrants may have children. This notion is congruent with findings in the hospital setting showing higher rates of patients with a lack of local language proficiency in reproductive than in geriatric or internal medicine departments in a Swiss hospital [22].

Our study has the advantage of focussing on the perceived language barrier instead of language proficiency in the local language, thus identifying only consultations where a lack of common language is actually relevant and some form of interpreting needed. This may be important when assessing unmet professional interpreter needs as a hospital based study from Switzerland [22] and a study focusing on primary care from Australia [38] have both demonstrated high rates of consultations in languages other than the local one, e.g., English, indicating that just focusing on local language proficiency may overestimate interpreter needs. Obviously, the language proficiency of both, physician and patient or, in the paediatric setting, the parents in such a language needs to be also sufficient.

The fact that the median rate for interpreter needs (median 15/year) lays lower than the rate of consultations with a relevant language barrier impeding direct communication with the patient or care-giver (median 24/year) indicates that for some consultations the presence of a professional interpreter is judged – probably correctly - unnecessary by physicians thanks to interpreting done by family, friends and staff and other circumstances not requiring the help of a professional interpreter. While physicians may underestimate the extent to which professional interpreters should ideally be used due to the lack of awareness, asking their perceived needs for such interpreters gives a closer idea to what they actually consider necessary and would potentially use if made available. Under certain circumstances professional interpreters may not be required. Establishing clear standards for when family, friends or staff can and when professional interpreters are to be used and research on the extent of “adequate” use of non-professional interpreters is warranted.

Participants in our study were of the opinion that communicating across language barriers in the absence of professional interpreters could have negative consequences on quality of care and lead to increased healthcare costs. The existence of an impact on quality of care [8, 26] and a cost saving potential through interpreter use [9] have previously been demonstrated predominantly in the hospital setting and are most likely relevant also for primary care. The extent of its occurrence in Swiss primary care warrants further investigation.

Insufficient use of professional interpreters is a phenomenon not unique to healthcare in Switzerland [15, 16, 18, 39–41] and may vary greatly between countries [41], but to our knowledge similar nation-wide information such as obtained by this study is lacking for other countries in Europe. The extent to which relevant language barriers are encountered in primary healthcare internationally is likely to vary according to the number of migrants with a linguistic background different to the host countries’ languages, their education levels and level of integration and therefore levels of proficiency in the local or commonly shared language (e.g., English) achieved. Similar studies may therefore be helpful in other host countries to evaluate existing interventions, to identify gaps and to provide policy makers with arguments and necessary information to implement interpreter policies for primary healthcare.

Awareness of the benefits of the use of professional interpreters, funding and policies in place, training of health professionals, but also the clinical situation and difficulties to assess language proficiencies for different degrees of complexity of consultations may influence interpreter use [18, 36]. Swiss primary care physicians are clearly aware of the need for professional interpreters. Further investigations are therefore needed into barriers to adequate professional interpreter use, such as funding, and to how the need for interpreters in general practice can best be addressed.

Considering that international research demonstrates some migrant groups experience difficulties accessing healthcare due to language barriers [1, 42], and poorer health [43], the importance of ensuring improved quality of communication in medical encounters, thus the use of interpreter services when needed, to achieve public health goals, is likely relevant in most receiving countries. It should include all types of migrants. Comprehensive interpreter policies including financial coverage should be considered for primary healthcare in Switzerland to address unmet interpreter needs demonstrated in this study.

## Conclusion

This is the first study to demonstrate the extent of the language barrier in Swiss primary care and potential negative consequences on quality of care causing potential harm for patients and extra health care costs. Professional interpreter use is clearly insufficient. Primary care physicians are aware of this and express their unmet professional interpreter needs. Further research is warranted on which elements hinder and which elements would enhance adequate use of professional interpreters in primary care. Our results indicate the urgent need for policies for a more equitable access to health also on the primary care level.

## Abbreviations

EU: European Union
FD: Family doctor/general practitioner
KHM: Kollegium für Hausarztmedizin; Collège de medicine de premier recours
MFE: Haus- und Kinderärtze Schweiz; Médecins de famille et de l’enfance Suisse
PCP: Primary care paediatrician
SSP: Swiss Society of Paediatrics
